# Mapping the effectiveness of the community tuberculosis care programs: a systematic review

**DOI:** 10.1186/s13643-023-02296-0

**Published:** 2023-08-03

**Authors:** Gabalape Arnold Sejie, Ozayr H. Mahomed

**Affiliations:** 1 Discipline of Public Health Medicine, University of KwaZulu, Natal, Durban, South Africa; 2 Department of Health Promotion and Education, Boitekanelo College, Gaborone, Botswana; 3 Dasman Diabetes Institute, Kuwait City, Kuwait

**Keywords:** Tuberculosis, Community tuberculosis, Cost-effectiveness, Treatment outcomes

## Abstract

**Background:**

Tuberculosis is a significant global public health threat, especially in countries with limited resources. To improve tuberculosis care, the World Health Organization emphasizes the importance of considering a TB patient’s journey across a variety of connected settings and facilities. A systematic review was conducted to map previously conducted studies to identify existing community TB implementation models, their effectiveness on cost, and treatment outcomes.

**Methods:**

Systematic search through various electronic databases MEDLINE, EBSCO (PsycINFO and CINAHL), Cochrane Library, EMBASE, WHO Regional Databases, gray literature, and hand-searched bibliographies was performed. Articles published in English between the years 2000 and 2022 with a substantial focus on community TB implementation models were considered for inclusion. Studies were excluded if the intervention was purely facility-based and those focusing exclusively on qualitative assessments. Two reviewers used standardized methods to screen titles, abstracts, and data charting. Included studies were assessed for quality using ROBINS-I and ROB 2. Analysis of study results uses a PRISMA flow diagram and quantitative approach.

**Results:**

A total of 6982 articles were identified with 36 meeting the eligibility criteria for analysis. Electronic medication monitors showed an increased probability of treatment success rate (RR 1.0–4.33 and the 95% CI 0.98–95.4) in four cohort studies in low- and middle-income countries with the incremental cost-effectiveness of $434. Four cohort studies evaluating community health worker direct observation therapy in low- and middle-income countries showed a treatment success risk ratio of up to 3.09 with a 95% CI of 0.06–7.88. (32,41,43,48) and incremental cost-effectiveness up to USS$410. Moreover, four comparative studies in low- and middle-income countries showed family directly observed treatment success risk ratio up to 9.07, 95% CI of 0.92–89.9. Furthermore, four short message service trials revealed a treatment success risk ratio ranging from 1.0 to 1.45 (95% CI fell within these values) with a cost-effectiveness of up to 350I$ compared to standard of care.

**Conclusions:**

This review illustrates that community-based TB interventions such as electronic medication monitors, community health worker direct observation therapy, family directly observed treatment, and short message service can substantially bolster efficiency and convenience for patients and providers while reducing health system costs and improving clinical outcomes.

**Supplementary Information:**

The online version contains supplementary material available at 10.1186/s13643-023-02296-0.

## Background

Tuberculosis (TB) is a notable health risk to current and future global health, with millions of people continuing to fall sick with the disease each year. In 2021, World Health Organization (WHO) projections indicated TB as the 13th leading cause of death and the second deadliest infectious killer after COVID-19 [1]. Globally, an estimated 9.9 million people fell ill with TB in 2020, a number that has been relatively stable in recent years [1]. The burden of disease varies enormously among countries, with the global average being around 127 cases per 100,000 population [1]. TB affects people of both sexes in all age groups; however, the highest burden is in men, who accounted for 56% of all TB cases in 2020. Geographically, most TB cases in 2020 were in the WHO regions of South-East Asia (43%), Africa (25%), and the Western Pacific (18%), with smaller percentages in the Eastern Mediterranean (8.3%), the Americas (3%), and Europe (2.3%) [1]. African region had the most TB burden countries with the highest TB incidence of 237 cases per 100,000 population [1]. The proportion of TB cases coinfected with HIV was highest in countries in the WHO African Region, exceeding 50% in parts of southern Africa [2].

The global TB treatment coverage was down from 72% in 2019 to 59% in the year 2020. Among the six WHO regions, treatment coverage was highest in Europe with a best estimate of 69% and lowest in the Eastern Mediterranean with a best estimate of 52% [2]. The treatment success rate for the new and relapse cases treated in the 2017 cohort globally was 85% [1]. Among the six WHO regions, the highest treatment success rates in 2017 of 91%, were in the WHO Eastern Mediterranean Region and the Western Pacific Region. The lowest rates were 76% in the WHO Region of the Americas and 78% in the European Region [1]. There were an estimated 1.3 million TB deaths among HIV-negative people in 2020, and an additional 214,000 deaths among HIV-positive people with about 83% of TB deaths among HIV-negative people occurring in the WHO African Region and the WHO South-East Asia Region [1]. The global reduction in the total number of TB deaths between 2015 and 2020 was 9.2%, about one quarter of the way to the End TB Strategy milestone of a 35% reduction by 2020 [2].

The WHO End TB Strategy envisions a TB-free world by the year 2035 [3]. In light of limitations inherent in prevailing tuberculosis care and the global urgency to improve TB care, the WHO emphasizes the importance of taking into consideration the journey of a TB patient through a series of interlinked settings and facilities [4]. One of the ways to do this is by decentralizing TB care beyond health facilities and harnessing the contribution of communities through the provision of effective community-based directly observed therapy (DOT) to TB patients at greatest socio-economic risk [3]. These patient-centered community-based care models are considered necessary to increase the capacity of the public health system to provide treatment to more TB patients and may address patients’ needs more successfully as care closer to home is easier to access, convenient, allows family support, and eliminates long and costly trips to a centralized primary health facility [5, 6]. However, despite the clear need, the documented cost-effectiveness of community-based TB activities and the tremendous efforts that have been expended in recent years, the program is not yielding the expected results. Treatment success rates in many countries are declining and remaining below global targets, resulting in increased healthcare expenditure and poor quality of life for the victims [1]. Projected trends indicate that a substantial strengthening of efforts to reduce TB incidence is needed if the WHO End TB strategy is to be met in countries with high TB incidence [7]. Whereas frameworks to accelerate the reduction in TB incidence can be reached by expert consensus, it is fundamental that these are underpinned by a robust and up-to-date evidence base for the effectiveness of specific interventions. Such an evidence base also allows for the harmonization of best-practice approaches to TB control [7]. The purpose of the systematic review is to aid the research in identifying previously conducted studies to identify what models of community TB implementation exist, their cost-effectiveness, and their effectiveness on patient outcomes. The systematic review will also assess the disadvantages and advantages of the different models of implementation in relation to cost effectiveness in low- and medium-income countries (LMIC). Although we acknowledge that there have been other systematic studies conducted in the past, their attention was primarily focused on one or two models with a small sample size, and our study broadened the scope to include more models of delivery; as a result, the findings of this review aim to comprehensively add to the body of knowledge in the area of community TB care implementation, inform policy, planning, and direct future research.

## Methods

The study was analyzed and reported in accordance with the Cochrane systematic review guidelines and the Preferred Reporting Items for Systematic Reviews and Meta-Analyses (PRISMA) to include constituents that resonate with the underpinnings of the systematic review methodology [8, 9]. Investigators developed the systematic review protocol including the eligibility criteria and the data abstraction tool. No formal protocol was published for this systematic review.

### Types of outcome measures

#### Primary outcomes

1. Identifying previously conducted studies to identify what models of community TB implementation exists.

#### Secondary outcomes

1. Any data pertaining to model cost-effectiveness and effectiveness on patient outcomes.
2. Assess the disadvantages and advantages of the different models of implementation in relation to the cost-effectiveness in low- and medium-income countries (LMIC).

### Literature search strategy

A comprehensive search strategy was developed using appropriate keywords, Medical Subject Headings (MeSH), and free text terms to maximize the retrieval of potentially relevant studies. The search was conducted across various electronic databases between the years 2000 and 2022: MEDLINE/PubMed, EBSCO (PsycINFO and CINAHL), Cochrane libraries, EMBASE, and WHO Regional Databases; Keywords used to search for these databases included but not confined to tuberculosis, community tuberculosis care, implementation models, cost-effectiveness, and treatment outcomes. During the search, keywords were separated by Boolean terms (AND, OR, NOT). We complemented these database search results with “gray literature,” including hand-searched bibliographies to identify any studies which may have been missed by the above search strategies.

The search for gray literature was conducted by compiling a list of potential organizations and academic institutions active in TB research. Government agencies, advocacy groups, private agencies, and non-governmental organizations were also considered. Publications were located on their identified organization website using either tab or page dedicated to publications and/or research findings or using the site’s search function. A list of key people, authors, and groups was contacted via mail. Moreover, social networks like ResearchGate and LinkedIn were used to locate others working in the topic area or to contact an author directly. Similarly, reference lists from relevant studies were scanned and identified authors were followed through social networks and emails.

### Screening and eligibility determination

Eligibility was based on the following inclusion and exclusion criteria:

**Table:** 

No	Inclusion criteria	Exclusion criteria
1	• Articles published in English	• Articles that were not published in English were excluded as the researcher will not assess them due to the language barrier
2	• Year of publications between the years 2000 and 2022	• Studies were excluded if the intervention was purely facility-based
3	• Literature with substantial focus on community TB implementation models including peer-reviewed journal articles, systematic reviews, scoping reviews, meta-analysis and rapid reviews, government and non-governmental organization reports, and academic dissertations. Unpublished manuscripts, conference abstracts, and theses	
4	• Research focusing on community TB implementation models in low-income and middle-income countries and whose conclusions and discussion demonstrate transferable findings to Botswana settings	
5	• All study designs were considered including quantitative and mixed-methods studies	Studies which focused exclusively on qualitative assessments

Studies obtained through database searches were exported to the Endnote library for further abstract and full article screening respectively. The Endnote library “find full text” option was used to automatically download PDFs of exported studies. A global search approach was utilized then contextual interpretation for low-middle income countries.

**Fig. 1:**
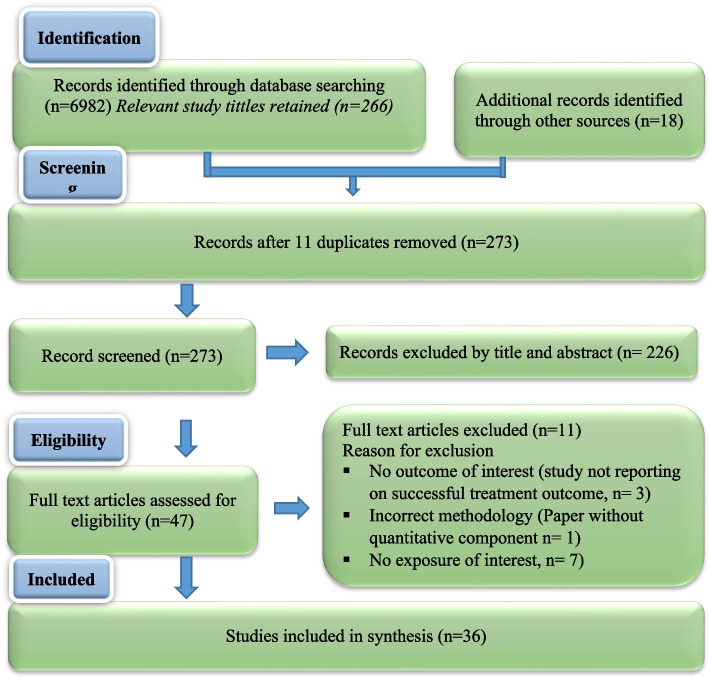
Preferred Reporting Items for Systematic and Meta-Analyses flow diagram (Adapted from Moher et al.) [11]

Using a two-reviewer system (with consensus for disagreements and conferral with a 3rd party adjudicator if a consensus was unable to be reached), all articles identified were screened by reviewing the title and abstract to remove all articles that clearly did not meet the eligibility criteria. The full text of the remaining articles was reviewed by two reviewers, and any ensuing discrepancies were resolved by discussion or the involvement of the third reviewer if a consensus was not reached by the first two reviewers. In accordance with recommendations by Levac et al. [10] after reviewing every batch of 20 to 30 publications, the reviewers meet to resolve any conflicts and ensure consistency with the research question and purpose. To capture and present the screening process, the Preferred Reporting Items for Systematic and Meta-Analyses flow diagram in Fig. 1 was used [9, 11].

### Methodological quality assessment

We assessed the risk of bias in randomized control trials (RCTs) in this review using the Cochrane risk-of-bias tool for randomized trials (RoB 2) according to the following domains: bias arising from the randomization process, bias due to deviations from intended interventions, bias due to missing outcome data, bias in the measurement of the outcome, and bias in the selection of the reported result [12]. Cluster randomized trials were assessed using the risk of bias for cluster randomized studies [8].

Observational studies were assessed using the risk of bias in the non-randomized studies-of-interventions (ROBINS-I) tool. The ROBINS-I assesses four broad areas: confounding, selection bias, information bias, and reporting biases [13]. The overall quality of the evidence for the primary outcome was assessed with the adapted GRADE approach [8]. Domains that may decrease the quality of the evidence are study design and implementation (risk of bias), inconsistency (heterogeneity), indirectness (inability to generalize), imprecision (insufficient or imprecise data), and publication bias across all studies that measure that particular outcome. The quality of the evidence on a specific outcome is based on the performance against six factors: study design, risk of bias, consistency, and directness of results, the precision of the data, and publication bias across all studies that measured that particular outcome. Two reviewers appraised each study independently and disagreements were resolved through discussion with a third reviewer.

### Charting the data and collating, summarizing, and reporting the results

A comprehensive data abstraction format in Microsoft Excel was developed collectively by the reviewers to extract predetermined variables. Prior to full extraction, the tool was tested with four articles and refined. Structuring this Excel sheet database involved selecting and defining data categories and subcategories. It was secured online so that involved reviewers will have access and can make updates freely. Bibliographic details, study design, intervention(s), comparison(s), measures of effect (risk ratios, or odds ratios with respective confidence interval) outcomes, study setting, and conclusions for the primary and secondary outcomes of interest were extracted. The geographic origin of the papers was categorized according to the World Bank country classification by economic level which includes low- and middle-income countries and high-income (developed) countries. This dataset was populated from each selected paper. This step was done iteratively as more familiarity of literature is gained and revisions were done as appropriate. This was purposively done to keep track of the studies included and excluded during the charting process of the systematic review. Two independent reviewers did data charting. The extracted data were extrapolated into a data charting form in a Microsoft Excel file depicting: the existing community TB implementation model as it relates to study designs used, type of models, their effectiveness on treatment outcomes, and cost-effectiveness. The data was analyzed using a quantitative approach to address the main aim and the specific study questions. Further to this, the study team scrutinized the meanings of the findings as they relate to the overall purpose of the study and discuss the implications for future research, practice, and policy. The goal of the systematic review is to provide an overview of the available literature, so all studies were included regardless of the quality assessment outcome. Information, including predetermined variables, is summarized descriptively. See Table S1 for the data extraction form.

### Model clinical effectiveness

The measure of effectiveness was based on sputum smear results at the end of the 2nd and 6th months of treatment. Patients with at least two negative smears including the smear at the 6th month were reported as cured. Patients who finished the treatment but did not have the 6th-month smear result were reported as treatment completed. We used treatment success rate (TSR)as a measure of effectiveness, which is a standard indicator used by WHO to measure program success. TSR was calculated as the sum of the number of TB patients who were cured and the number of TB patients for which treatment was completed divided by the total number of smear-positive cases reported, expressed as a percentage.

### Models economic viability/cost-effectiveness

Cost-effectiveness was determined by dividing the total cost for each model by the treatment success rate at 6 months. Final costs were estimated and costs per patient cured were compared with the aim to determine the average cost, and the marginal or incremental cost for an additional unit of health benefit when choosing between two models, the incremental cost-effectiveness ratio and average cost-effectiveness, was used. The incremental cost-effectiveness ratio was defined as the difference in total costs between intervention and control divided by the difference in the number of patients with sputum conversion and those who completed treatment between intervention and control.

## Results

### Results of the search

A total of 6982 references were identified from the bibliographic search. Of this, the screening of titles yielded 284 studies. After removing duplicates, we identified 273 potentially relevant references; 226 were excluded based on title and abstracts, leaving 47 studies that were acquired in full text or study report with available information for further evaluation. After conducting a full-text review, thirty-six studies were included in our systematic review. A hand-search of references of the included studies revealed no further relevant publications. We used the PRISMA checklist for the assessment of meta-analysis guideline compliance (Fig. 1).

### Study characteristics

**Fig. 2:**
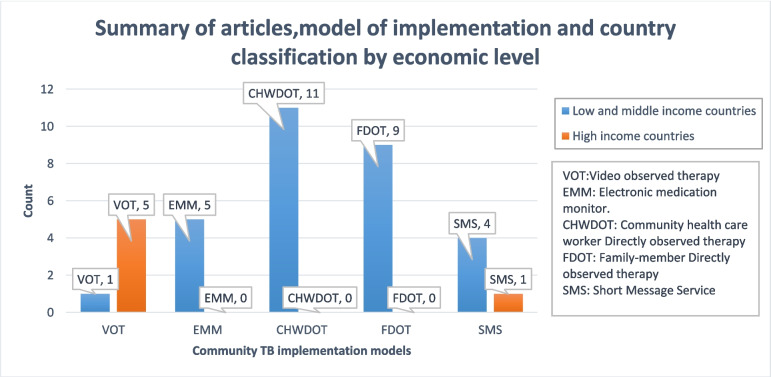
Summary of articles showing model of implementation and country classification by economic level

The thirty-six included studies were published between 2000 and 2022. They consisted of eleven RCTs [14–24], four cluster randomized control trials [25–28], nineteen seventeen cohort studies [29–47], one record review [48], and one quasi-trial [49]. All thirty-six had a control group and provided estimates of effect: eleven evaluated community health care worker direct observation therapy (CHWDOT) [14, 19, 24–27, 29, 31, 33, 38, 39], nine evaluated family direct observation therapy (FDOT) [21–23, 28, 37, 40, 41, 48–50], five evaluated short message service reminders (SMS) [15–18, 51, 52], six examined video observed therapy (VOT) [20, 32, 34, 35, 43, 44], and five examined the electronic medication monitors (EMM) [30, 42, 45–47] (See Fig. 2). Substantive descriptions of the included studies including intervention evaluated can be seen in Tables S2 and S3.

Studies were excluded from the analysis for any of the following reasons: No exposure of interest, no outcome of interest, qualitative studies, articles available only in an abstract form, case reports, anonymous reports, studies reporting data purely on facility-based TB, and articles that are not in English because of unfulfilled the inclusion criteria [53–63] (Table S4).

### Risk of bias assessment

**Fig. 3:**
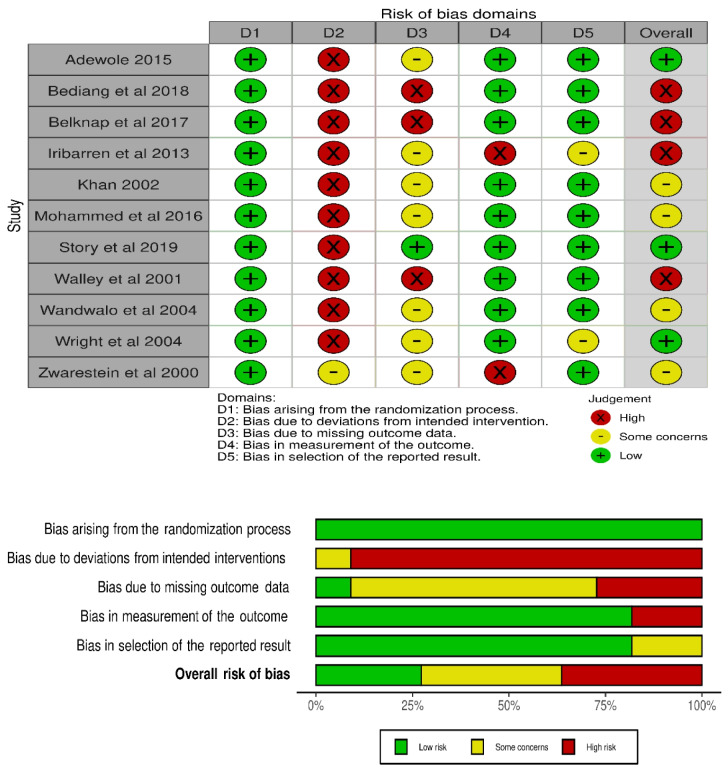
The risk of bias assessment of included papers using the ROB 2 tool for randomized studies

**Fig. 4:**
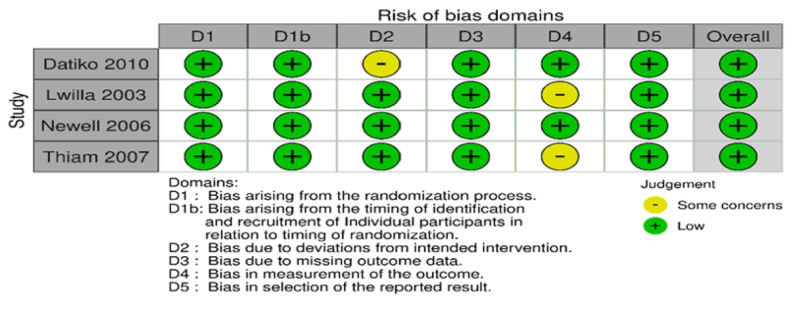
The risk of bias assessment of included papers using the ROB 2 tool for cluster-randomized studies

Quality assessment and risk of bias in the randomized trials reviewed are shown in Fig. 3. We found low selection bias, as all eleven publications of randomized trials provided information about the processes of random sequence generation and/or allocation concealment in the studies. Overall, there was high bias of performance across the trials. Detection/outcome measurement bias was high for two trials since the assessors were aware of the intervention received by study participants. Studies varied with respect to attrition bias. We also found low reporting bias in nine of the trials. For clustered RCT, a risk of bias 2 for the clustered tool was used, and four studies were evaluated with the overall risk of bias being low (Fig. 4).

**Fig. 5:**
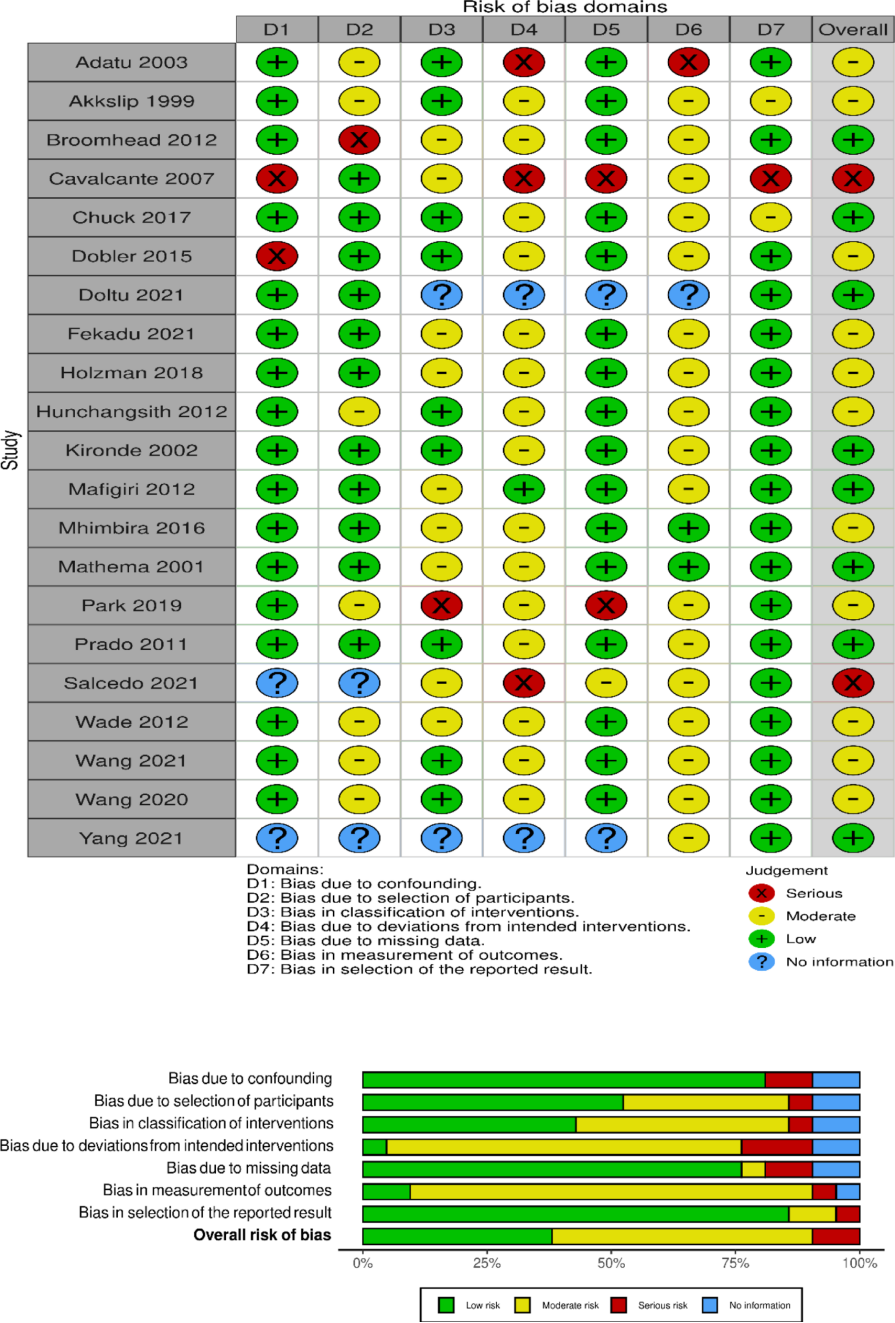
The risk of bias assessment of included papers using the ROBINS-I tool for non-randomized studies

Twenty-one studies included were observational, and methodologic quality in these studies was assessed using the Cochrane risk-of-bias in Non-randomized Studies of Interventions (ROBINS-I) scoring system [13]. ROBINS-I views each study as an attempt to simulate an ideal randomized trial that is expected to answer a particular clinical problem. Seven domains were investigated for the potential risk of introducing bias and that is judged with the use of signaling questions. Overall, the risk of bias was moderate in most papers, which is understandable as most studies were non-randomized and had a retrospective design, and as such are subject to confounding and a range of other biases (Fig. 5).

### Existing community TB care models

In this review, we identified five models of implementation to form the basis of our analysis. These models include video-observed therapy (VOT), electronic medication monitors (EMM), community health care worker directly observed therapy (CHW DOT), family-member directly observed therapy (FDOT), and short message service (SMS). Explanations of the model’s implementation are discussed below.

### Video-observed treatment (VOT)

Six studies reported on this model, and the model can be utilized both asynchronous and synchronous. The asynchronous video-observed therapy (VOT) application enables participants to record themselves swallowing each treatment dose and send videos for review by a DOT worker. Each recorded dose is automatically date- and time-stamped, encrypted, and uploaded to a secure server over a cellular or wireless network. Once the data is received by the server, the smartphone application deletes videos from the device to prevent unintentional disclosure of participant information and conserve device memory. Videos are stored on the smartphone in a manner that prevented viewing, editing, resending, or deleting them to protect participant privacy and ensure video fidelity. The asynchronous design allows participants to take their medications regardless of network connectivity (e.g., while traveling) because videos are uploaded automatically whenever cellular or WiFi connections are established. An application status screen allows participants to see when videos were uploaded or pending [20, 34, 64]. This intervention can also be accessed using a live video (synchronous video-observed therapy), and here the VOT worker and patient pre-arrange a schedule for the VOT calls. The VOT worker receives calls using a webcam-equipped computer. The patient shows and names each pill in front of the camera before swallowing it. To demonstrate that the pills had been swallowed, the patients open their mouths in front of the camera and engage in conversation with the VOT worker for several minutes. If any side effects are reported, a physician will be connected by video or audio to provide medical advice. Each VOT session is documented in the electronic medical record (EMR) system. Missed VOT appointments are followed up by phone calls and, if these are unsuccessful, home visits. The VOT worker records the start and end time of each session, including the time it took to document the session, in the EMR and the VOT database [32, 34].

### Electronic medication monitors (EMM)

Five studies reported on automated electronic devices that monitor and store adherence records combined with audible reminder alarms. This model consists of a device that attaches to the standard pill bottle or blister pack and records/sends an SMS every time the patient opens the bottle to a Web-based application indicating the patient has taken his or her medication. During each time the patients visited TB-designated hospitals at the county level, doctors generate the adherence report by connecting the EMM to an offline software program on their computers. Appropriate measures will then be taken depending on number of doses missed [30, 42, 45].

### Community health care worker directly observed therapy (CHW DOT)

Eleven studies reported on community health care worker directly observed (CHWDOT), and the community health care worker was defined as someone living in the same village as that of the patient and observed the patient daily. In this model of implementation, the CHWDOT observers who volunteered to work are interviewed and selected by the village leaders and trained to supervise the patient’s intake of TB medications. The CHWDOT observers are visited fortnightly by the TB health worker in charge of the nearby health facility. During house visits, the health workers monitor adherence to treatment by checking the treatment card and counting the pills remaining from the patient’s monthly drug supply. During the whole course of treatment, patients under CHWDOT visit the health facility monthly for drug collection. Drugs are collected from a nearby health facility by a patient or her/his CHWDOT observer, depending on the condition of the patient [24, 26, 27, 29, 31, 33, 38, 39, 65].

### Family-member directly observed therapy (FDOT)

Nine studies reported on family DOT strategy in which anti-TB medications are administered at home under the supervision of an adult household member [66, 67]. There are no specific criteria related to the education or occupation of the family member to qualify as a DOT provider. If clients do not want to have a family member provide DOT, then they are considered ineligible for the intervention. In such cases, a family supervisor was assigned to the client. The eligible family member who wished to become the DOT provider is given onsite training (at home) by government supervisory staff. The training focuses on the DOTS strategy, the treatment process and its duration, the role of DOT supervisors in ensuring TB treatment completion, and the side effects of anti-TB medications. The family member collects drugs fortnightly from the health facility most convenient for him or her. As per national guidelines, there would be two treatment cards for each patient: the original treatment card with the community DOT provider and a duplicate treatment card at the health center which is updated fortnightly by the government supervisory staff. All participants are later visited by either a medical officer or a treatment supervisor assigned to their respective areas. This step is taken to verify whether each client is receiving family DOT according to national operating procedures. Treatment monitoring is done by following up with the client as per national guidelines (clinical/bacteriological assessment at the end of 2 months and at the end of treatment) [21–23, 28, 40, 41, 48, 50, 66, 67].

### Short message service reminders (SMS)

Five studies evaluated the SMS reminders directly observed treatment, and this intervention utilizes a daily medication reminder system for TB patients. Once a patient is enrolled, the system sends daily SMS reminders scheduled at a time that the patient specified during enrolment. The messages include a motivational message followed by a reminder to patients to reply via SMS to indicate that they have taken their medication. Messages are sent every day throughout the full duration of patients’ treatment. To maintain the patients’ attention and interest, these messages are changed every 2 weeks. If a patient did not reply, additional reminders are sent, and the team would follow up to confirm that the messages are being received [15–18, 68, 69].

### Model clinical effectiveness

#### Video-observed treatment (VOT)

Two cohort studies conducted in developed countries of Australia and the USA in 2012 and 2017 reported a VOT treatment completion risk ratio ranging between 0.99 and 1.47 (95% CI 0.93–2.25) when compared with in-person DOT [32, 44]. Another cohort study in the low- and middle-income country of Moldova in 2021 reported a VOT treatment success risk ratio of 0.07 (95% CI 0.0–0.5) compared with facility DOT [34]. One RCT in a high-income country of the UK conducted in 2019 reported a higher proportion of treatment completion rates with VOT compared with in-person DOT (OR 2.52, 95% CI 1.17–5.47), but the effect on treatment completion rates was not statistically significant [20]. These results suggest that VOT is marginally effective on treatment outcomes in low-income countries but more effective in high-income countries.

#### Electronic medication monitor (EMM)

Four cohort studies from low- and middle-income countries of China, Morocco, and South Africa between 2012 and 2021 evaluated electronic medication monitors against DOT and self-administered treatment (SAT). The results of these studies showed EMM treatment success ratio ranging from 1.0 to 4.33, and the 95% CI fell within 0.98–95.4 values all with no statistically significant effect on treatment success [30, 42, 45, 46], suggesting that electronic medication monitor is effective in treatment outcomes, especially in countries with lower economies.

#### Community health care worker directly observed therapy (CHWDOT)

Four cohort studies conducted in low- and middle-income countries of South Africa, Uganda, Brazil, Kampala, and Mongolia between the years 2002 and 2015 evaluating community health worker delivered DOT against family DOT and facility or family-based DOT showed CHWDOT treatment success risk ratio ranging between 0.29 and 3.09 with 95% CI falling between 0.06 and 7.88 [31, 33, 38, 39]. Of these four studies, only one study in Uganda did not find the difference in the treatment success rate between CHWDOT and facility-based DOTS (OR = 0.29; 95% CI 0.06 to 1.34) [39]. Three RCTs also from countries of the low- and middle-income (Tanzania, South Africa, and Senegal) between 2000 and 2007 reported a CHWDOT treatment success risk ratio of 1.18–1.7 (95% CI 0.1–34.5) [24, 26, 27].

#### Family-member directly observed therapy (FDOT)

Four RCTs all from low- and middle-income countries of Tanzania, Eswatini, Nepal, and Pakistan evaluated family DOT against community health care workers, self-administered treatment, and facility DOT between 2001 and 2006 showed odds ratios ranging from 1.03 to 1.10 with 95% CI falling between 0.41 and 1.72, all without statistically significant effect on FDOT treatment completion, success, and cure rate [21–23, 28]. Four comparative studies (quasi-trial, retrospective cohort analysis, and records’ review) still from low- and middle-income countries of Brazil, Thailand, Nepal, and Tanzania between 2001 and 2016 showed no statistically significant effect on FDOT treatment success with risk ratio ranging between 0.94 and 9.07 and the 95% CI of 0.92–89.9 when compared with health facility DOT, self-administered treatment, and community health care worker DOT. Of these four, one study in Tanzania reported a low risk ratio to treatment success with the other three having a risk ratio of above one [40, 41, 48, 50].

#### Short message service reminders (SMS)

Four RCTs [15, 16, 18, 68] evaluating SMS as medication reminders showed no statistically significant effect on treatment completion when compared with the local standard of TB care. In three of these from the low- and middle-income countries of Cameron, Argentina, and Pakistan conducted between the years of 2013 and 2018 [15, 18, 68], the SMS group risk ratios for completion, success, or cure ranged from 1.0 to 1.45, and the 95% CI fell within these values in all three trials. One multinational RCT conducted in Spain, Hong Kong, the USA, and South Africa reported 76.4 vs 85.4% (95% CI 71.3–80.8%) SMS treatment completion compared to facility DOT [16]. This result shows that SMS DOT is an effective intervention for successful treatment outcomes.

### Model economic viability/cost-effectiveness

#### Video-observed treatment (VOT)

Four observational studies in high-income countries of Australia and the USA between 2012 and 2021 evaluated the cost-effectiveness of this model. Three of these have shown incremental cost-savings ranging between $1391 and $2226 when comparing VOT with using in-person DOT and VOT was therefore the preferred cost-effective option [35, 36, 43]. Another retrospective cohort design in Australia comparing patients receiving direct observation by home videophone with patients receiving this service in person has shown the incremental cost-effectiveness ratio (ICER) to be AUD$1.32 (95% CI $0.51–$2.26) per extra day of successful observation with VOT [44]. The video service used less staff time and became dominant if implemented on a larger scale and/or with decreased technology costs.

#### Electronic medication monitor (EMM)

Two observational studies in low- and middle-income countries of South Africa and Morocco in 2012 and 2021, respectively, evaluated cost-effectiveness under this model. One study in South Africa comparing the costs and health outcomes of the DOTS-SIMpill cohort with DOTS-only controls has shown a positive return on investment (ROI of 23% over the 5-year period) for the DOTS-SIMpill cohort based on improved health outcomes and reduced average cost per patient [30]. Another study conducted in Morocco evaluating the costs and cost-effectiveness of a Medication Event Monitoring System (using a smart pillbox with a web-based medication monitoring system) for tuberculosis management against standard of care (SoC) has shown the ICER of $434/DALY averted for managing drug-susceptible TB patients by MEMS relative to SoC; thus, MEMS is considered cost-effective in Morocco [47].

#### Community health care worker directly observed therapy (CHWDOT)

Three randomized control trials evaluating the cost-effectiveness of community health care worker DOT in low- and middle-income countries of Ethiopia, Pakistan, and Nigeria between 2002 and 2015 have reported in favor of community health care worker DOT with incremental cost-effectiveness ratio ranging between 16.3 and 410 US$ compared to health facility-based and self-administered DOT [14, 19, 25]. The community health worker became the most cost-effective approach.

#### Family-member directly observed therapy (FDOT)

Two observational studies in low- and middle-income countries of Brazil and Thailand conducted in 2011 and 2012 evaluated family DOT model cost-effectiveness, they all reported in favor of the model when compared with community health worker DOT and health care worker and Self-administered DOT. A study in Brazil has shown the guardian supervised/family DOT cost on average to be US$398 per patient cured. This figure was US$ 260 (39%) lower than its equivalent for CHW-supervised DOT (US$657) resulting in a saving of US$1.0095 per additional patient cured [50]. A study in Thailand has reported cost savings associated with family-member DOT (− I$9 million [95% uncertainty interval − I$12 million to − I$5 million]) with 9400 DALYs averted, ICER I$1100 dominant to I$1300 indicating that family-member DOT is a cost-saving intervention [37].

#### Short message service reminders

Two observational studies conducted in low- and middle-income countries of Thailand and Brazil in 2012 and 2018 have shown SMS intervention to be cost-effective when compared with SAT. An economic study in Thailand on SMS use in TB care demonstrated an incremental cost-effectiveness ratio of 350 “international dollars” per disability-adjusted life year [37]. Another decision analysis model developed to simulate cohorts who initiate TB treatment in Brazil compared SMS intervention has shown that among persons from the general population with latent TB infection, SMS was the most cost-effective intervention against SAT with the incremental cost (95% UR) of USD 164 (USD 29 saving to USD 362 cost) per DALY averted and USD 814 (USD 137 saving to USD 1781 cost) per TB case prevented. SMS cost USD 1000 per DALY averted and USD 4483 per TB case prevented compared to SAT [52].

## Discussion

Community TB care interventions are increasingly used to support TB treatment in diverse settings globally. This analysis is set to examine the potential cost and impact of various community TB care interventions/models as applied under program conditions globally. It provides preliminary insights into the potential impact and cost of several approaches to TB treatment support in this context. We estimated effectiveness and substantial cost savings with VOT, medication monitors, community health worker DOT, family DOT, and SMS including savings to patients and their families, compared to other treatment options. While evidence remains incomplete, and generalizability limited, the studies reviewed suggest these interventions may improve efficiency, save money, and reduce burden on patients and healthcare workers.

A cohort study in Moldova reported a protective facility DOT over VOT whereas studies in the USA, UK, and Australia suggested that treatment outcomes were improved compared with in-person DOT, with markedly reduced health system costs. These results suggest that VOT is ineffective in improving treatment success in low-income countries but more effective in high-income countries. This could be mainly due to the accessibility of digital platforms in developing countries posing a challenge for model efficacy. Therefore, until VOT becomes cheaper, it will probably be substantially less cost-effective for supporting treatment in low-income settings while in settings where digital solutions cost less and/or are easier to implement and use than the standard of care, VOT may be a beneficial alternative [52, 70].

Electronic medication monitoring system improved the TB treatment success rates in this review. Over time, the EMM group showed a higher treatment success rate and cost savings compared to the standard conventional TB treatment over a 6-month period. EMM is expected to contribute to the effectiveness (treatment outcomes and cost) of the TB case management strategy based on its convenient and effective monitoring of medication in low- and middle-income countries.

This review finding has shown that community-based DOT produced outcomes that were equivalent and or superior to the other treatment options for TB patients suggesting that CHW can effectively dispense anti-TB medication and community participation should be encouraged. The implication of this in high TB burden settings is that community-based TB treatment is an effective and viable option that can supplement other modes of treatment delivery. Furthermore, community-based TB treatment delivery has been found to be cost-effective, and it is a low-cost technology that can easily be adapted to diverse areas of need and appropriate CHW recruited according to availability in each contextual setting.

Family-supervised DOTS was more effective and less costly than other forms of DOT delivery. Implementation of family-supervised DOTS exceeded the quality of patient outcomes from other treatment options. In cost-effectiveness parlance, the results indicate that guardian-supervised DOTS was the dominant strategy. A possible explanation is that direct observation by family members incurs little inconvenience and negligible costs to the patient, as patients, once well enough, are not constrained in continuing their normal work. In addition, there is less potential for stigma [23].

This systematic review showed a paucity of high-quality evidence concerning the effect of mobile-phone messaging on anti-TB treatment success. All the studies in this review were from low- and middle-income countries. Mobile-phone messaging showed a modest effect in improving TB treatment success and cost saving. Results from this review concurs with evidence from meta-analyses of RCTs in other disease settings which have shown a positive effect of two-way SMS on treatment outcomes, which suggests that compared with DOT, SMS reminding regularly could significantly increase the patients’ successful TB treatment [69]. The most likely reason is that besides daily drug intake schedules, the SMS group patients could receive extra frequent prompts and health information, which gradually propel patients to practicing good habits and health awareness [68].

### Limitations

Our review has several limitations. We focused on quantitative comparisons of clinical outcomes, as these are fundamental to the evidence base. For this reason, we have not provided a detailed review of studies that focused exclusively on qualitative assessments. Given the marked heterogeneity of study designs, endpoints, and settings, we were unable to pool the estimates of effect and could only summarize findings as reported from each of the studies. The other weakness of this study was the necessity of using a retrospective cohort comparison between the intervention and control groups. While matching was undertaken for the available data, other confounding factors may have existed and the effect of these is unknown.

## Conclusions

In conclusion, this review illustrates that community-based TB interventions such as CHWDOT, FDOT, SMS, and EMMs play a successful role in improving the treatment success rate in low- and middle-income countries. Scarce resources can be conserved by managing larger numbers of TB patients with the same number of staff and thus cost-effective. Moreover, community-based DOT can substantially bolster efficiency and convenience for patients and providers thus saving costs and improving clinical outcomes.

### Supplementary Information

**Additional file 1: Table S1.** Data extraction form. **Table S2.** General information for included studies on community-based TB interventions and their impact on treatment outcomes (28 studies). **Table S3.** General information for included studies on community-based TB interventions and their cost effectiveness (12 studies). **Table S4.** List of excluded studies along with reasons for exclusion. Prisma checklist.

## Data Availability

All data generated or analyzed during this study are included in this published article [and its supplementary information files].

## Abbreviations

CHWDOT: Community health care worker directly observed therapy
DALY: Disability-adjusted life years
DOT: Directly observed therapy
EMMs: Electronic medication monitor
FDOT: Family-member directly observed therapy
ICER: Incremental cost-effectiveness ratio
RCT: Randomized control trial
RoB 2: Cochrane risk-of-bias tool for randomized trials
ROBINS-I: Risk of bias in non-randomized studies of interventions
SAT: Self-administered treatment
SMS: Short message service reminders
TB: Tuberculosis
VOT: Video-observed treatment
