# Supplementary material for: Mapping the effectiveness of the community tuberculosis care programs: a systematic review
Source: Syst Rev. 2023 Aug 3;12:135. doi: 10.1186/s13643-023-02296-0 (PMC10399107; doi:10.1186/s13643-023-02296-0)
Supplement: Supplementary file 1 — Additional file 1: Table S1. Data extraction form. Table S2. General information for included studies on community-based TB interventions and their impact on treatment outcomes (28 studies). Table S3. General information for included studies on community-based TB interventions and their cost effectiveness (12 studies). Table S4. List of excluded studies along with reasons for exclusion. Prisma checklist. [file 13643_2023_2296_MOESM1_ESM.docx]

Table 1: Data extraction form

| Author and date of publication Study title Aim(s) of the study or research questions Study design |  |
| --- | --- |
| Study setting (urban/rural) |  |
| Population |  |
| - Sample size |  |
| Methodology/intervention Type of Intervention and outcomes |  |
| Most relevant finding Most significant finding |  |
| Other key findings Study limitations and implications |  |
| Interpretations and conclusions from the authors |  |
| Comment/s |  |

Table 2: General information for included studies on Community Based TB interventions and their impact on treatment outcomes (28 studies).

| # | **First author [ref.]** | **Objective** | **Intervention** | **Control** | **Design** | **Region** | **Study outcome (definition)** | **Findings in control group/standard care %** | **Findings in intervention group%** | **Measure of effect (95% CI)** |
| --- | --- | --- | --- | --- | --- | --- | --- | --- | --- | --- |
| 1 | Bediang 2018 | To evaluate the effectiveness of SMS reminders as an adjunct to DOT in improving TB treatment adherence and success. | SMS | selective DOT (once weekly or monthly distribution of drug) with no SMS | Randomised, concealed, single-blinded controlled trial | Yaoundé,Cameroon | Treatment success rate (assessed at 5th month) | 75 | 81 | RR: 1.45 (0.81–2.56) |
| 2 | Iribarren 2013 | To evaluate the acceptance and feasibility of a patient-based text intervention to promote their adherence to TB treatment | SMS | Self administered treatment (SAT) without intervention(SMS) | Randomised, concealed non-blinded controlled trial | Buenos Aires,Argentina | Treatment success: cured or completed treatment | 53 | 77 | RR: 1.06 (0.87–1.28) |
| 3 | Mohammed 2016 | To measure the impact of a two-way SMS reminder system on TB treatment outcomes | SMS | DOT without SMS | Randomised, non-blinded controlled trial | Karachi, Pakistan | Treatment success: cured or completed treatment | 83 | 83 | RR: 1 (0.96–1.04) |
| 4 | Belknap 2017 | To compare treatment completion and safety of once-weekly isoniazid and rifapentine by self-administration versus direct observation. | SMS | Facility DOT | Individual RCT, blinding info NA | US, Spain, Hong Kong, and South Africa | Treatment completion | 85.4 | 76.4 | (CI, 71.3% to 80.8%) |
| 5 | Wang 2020 | To assess the relative differences in unfavourable outcomes and deaths among those started on EMM at baseline (within first month of diagnosis) when compared to SAT alone. | Electronic medication monitors/ | SAT | Cohort study using secondary data. | China | Treatment success: cured or completed treatment | 67 | 63 | ARR:1.00,95%CI (0.98, 1.03) |
| 6 | Wang 2021 | to study whether introducing EMM under programme settings and increasing its coverage results in improved TB treatment outcomes at the county level in China. | EMM | Community health worker | Longitudinal ecological study (stepped-wedge design) involving county-level aggregate secondary programmatic data | China | Treatment success: cured or completed treatment | 94 | 94.9 | (95% CI: 94.4–95.4),P=0.809 |
| 7 | Park 2019 | to investigate the effectiveness of the MEMS program by analyzing and comparing the initial data of the MEMS program with the conventional TB control program. | Medication Event Monitoring System (MEMS) | Direct supervision for the initial one to two weeks and self-administered treatment (SAT) for the remaining periods | Retrospective cohort study | Morocco | Treatment success: cured or completed treatment | 79.5 | 93.2 | (OR): 4.33, 95% (CI): 2.13–8.81, p < 0.001) |
| 8 | Broomhead 2012 | The return on investment (ROI) for utilizing the SIMpill electronic treatment adherence solution as an adjunct to directly observed treatment short-course (DOTS) is assessed using data from a 2005 pilot of the SIMpill solution among new smear-positive tuberculosis (TB) patients in the Northern Cape Province. | Medical adherence support (MAS) system | DOTS-only controls | A retrospective analysis comparing the costs and health outcomes of the DOTS-SIMPill cohort with DOTs-only controls | South africa | Cure | 72 | 100.00 | RR 2.32 (95% CI 1.60 – 3.36) |
| 9 | Chuck 2017 | To determine whether video technology for remote observation of patients on anti-TB treatment (VOT) is as effective as in-person DOT | VOT | In person DOT (Hospital/clinic or community) with no VOT | Prospective cohort study | New York, USA | Treatment completion | 97 | 96 | RR: 0.99 (0.93–1.05)P=0.63 |
| 10 | Wade | To assess the effectiveness related to patient compliance, cost effectiveness, acceptability and sustainability of video-based DOT | VOT | DOT by a drive around service or by clinic attendance | A retrospective cohort design | Adelaide, South Australia | Treatment completion | 33 | 48 | RR: 1.47 (0.96–2.25) |
| 11 | Story At al | to compare treatment observation with asynchronous VOT versus in-person DOT for supporting treatment adherence in patients with active tuberculosis | VOT | DOT based in a clinic, community (eg, pharmacy or hostel), or home setting | Analyst-blinded, randomised controlled | UK | Treatment completion | 63 | 77 | OR 2.52 (1.17-5.47 )P=0.0.19 |
| 12 | Doltu 2021 | TO compare adherence and short and long-term TB treatment outcomes for TB patients who experienced asynchronous Video Observed Treatment (aVOT) during three months of outpatient treatment versus Directly Observed Treatment (DOT) in operational conditions. | VOT | Facility based DOT | Cohort study combining data from Randomised control trials | Moldova,Chisinau | Cured or treatment completion | 82 | 98.8 | RR: 0.07(0.0-0.5) |
| 13 | Newell 2006 | To assess the success rates of the two DOTS strategies (FDOT&Community DOT) | Family DOT | Community health worker | Cluster randomised control trial | Nepal | Treatment success | 85 | 89 | OR: 0.67 (0.41- 1.10) |
| 14 | Prado 2011 | To compare the cost ans outcomes associated with community health worker supervised and guardian supervised directly observed treatment | Family DOT | Community health worker | Comparative study/quasi trial | Vitoria,Espirito santo state,Brazil | Cure | 83 | 98 | OR 9.07(1.12-73.18,P=0.04 |
| 15 | Walley 2001 | To assess the effectiveness of different packages for tuberculosis treatment under operational conditions | Family DOT | SAT | Randomised trial | Pakistan | Cure or treatment completion | 65 | 62 | OR: 1.08(0.67-1.72) |
| 16 | Akkslip 1999 | To evaluate the effectiveness of a programme offering the option of direct observation of treatment (DOT) by a supervised family member. | Family DOT | Self-administered | Review of patient records | Yasothorn Province, Thailand | Cure | 70.9 | 85.2 | 85.2%, 95% confidence interval [CI] 80.5– 89.9) |
| 17 | Wright 2004 | To evaluate and compare the ffectiveness of each method of patient supervision | Family DOT | Community health worker | RCT | Swaziland | Cure or treatment completion | 68 | 66 | OR 1.10(0.87-1.40 |
| 18 | Mathema 2001 | To assess TB treatment supervision strategies and outcomes | Family DOT | Self administered DOT | cohort study | Nepal | Cure | 57 | 34 | 57% (95%CI 48.8–64.0) |
| 19 | Wandwalo 2004 | To evaluate the effectiveness of community-based direct observation of treatment (DOT) using guardians and former TB patients compared to hospital-based DOT in an urban setting in Tanzania. | Family DOT | health facility based | Randomised controlled trial. | [Dar es Salaam  Tanzania](https://en.wikipedia.org/wiki/Dar_es_Salaam) | Cure or treatment completion | 83 | 85 | RR 1.03[0.96,1.1] |
| 20 | Mhimbira 2016 | to assess TB treatment outcomes in home-based and facility-based DOT under programmatic conditions in the high TB incidence country Tanzania | Family DOT | health facility based | A retrospective analysis of a cohort | Tanzania | successful treatment outcome | 86.4 | 81.6 | RR 0.94, 95% CI: 0.92–0.97 |
| 21 | Zwarenstein 2000 | To compare successful tuberculosis treatment outcome rates between self supervision, supervision by lay health worker (LHW), and supervision by clinic nurse | Lay health worker/Community health worker | Clinic nurseself supervision | Unblinded randomised control trial | Capetown,South africa | Cure or treatment completion | 57 | 74 | LHW vs. clinic nurse: risk difference 17.2%, 95% confidence interval [CI] −0.1–34.5; |
| 22 | Lwilla 2003 | To assess whether in new patients with smear-positive pulmonary tuberculosis (PTB) the smear conversion rate at 2 months and treatment outcome (specifically cure rates) were similar using CBDOT and IBDOT | Community health worker | Institutional based DOT | Unmasked cluster randomised control trial | Tanzania | Cure | 49 | 53 | OR: 1.58(0.32-7.88,P=0.57 |
| 23 | Kironde 2002 | To explores the feasibility of community participation in a high-burden TB programme in a resource-limited setting and attempts to establish how supervision of TB treatment by lay volunteers compares with other methods of TB treatment delivery | Community health worker | Clinic based and self administered | Prospective study involving patients with confirmed pulmonary TB who were followed-up over a one-year period. | Northern Cape province of South Africa. | Cure or treatment completion | 70 | 72 | RR=1.04[0.94–1.16], p=0.435) |
| 24 | Cavalcante 2007 | To compare community based direct observation treatment for tuberculosis using community health workers with clinic based DOT | Community health worker | Self administered treatment | Longitunal cohort study | Rio Dejenairo,Brazil | Cure or treatment completion | 75.8 | 90.4 | OR:3.09(1.99-4.81),P<0.001 |
| 25 | Mafigiri 2012 | To examine modified CB-DOTS as ‘proof of concept’ by identifying how the patients’ social support system may contribute to treatment seeking through task shifting in a high-prevalence urban setting of Kampala, Uganda. | Community health worker | Clinic based | Combination of qualitative and quantitative methods | Kampala, Uganda | Cure or treatment completion | 89 | 70 | (OR 0.29; 95% CI: 0.06-1.34) |
| 26 | Thiam 2007 | To assess the effectiveness of a contextualized intervention strategy aimed at improving patients' adherence to treatment and to evaluate its impact on TB control in a resource-poor country in Africa with prevalent TB infection. | Community health worker | Facility based | Cluster randomized controlled trial (RCT) | Senegal | Cure or treatment completion | 76 | 88 | [RR], 1.18; 95% [CI], 1.03-1.34). |
| 27 | Adatu 2003 | To measure the effectiveness and acceptability of community-based tuberculosis (TB) care using the directly observed treatment, short-course (DOTS) strategy for TB control. | Community health worker | Facility based | Comparative study | Uganda | Cure or treatment completion | 56 | 74 | (RR 1.3, 95%CI 1.2-1.5, P < 0.001) |
| 28 | Dobler 2015 | to determine the effectiveness of community-based DOT compared to traditional clinic-based DOT in Ulaanbaatar, the capital of Mongolia. | Community health worker | Family DOT | Retrospective cohort study | Mongolia | Cure or treatment completion | 83.2 | 93.6 | OR2.95 CI1.85-4.71 P<0.001 |

Table 3: General information for included studies on Community Based TB interventions and their cost effectiveness (12 studies)

| # | **First author[Ref]** | **Objective** | **Intervention** | **Control** | **Study design** | **region** | **Cost findings in control group** | **Cost findings in intervention group** | **DALYs reduced** | **incremental cost-effectiveness ratio** | **Estimated cost savings** |
| --- | --- | --- | --- | --- | --- | --- | --- | --- | --- | --- | --- |
| 1 | Fekadu 2021 | To estimate the outcomes of pandemic-related DOT suspension and the cost-effectiveness of video-observed therapy (VOT) during the pandemic. | VOT | SAT | Decision analytical model | USA | US$14334 | US$12537 | 0.4299 | -3690 | US$1871 (95% CI US$1797– 1944; P<0.01) |
| 2 | Wade 2012 | To evaluate the clinical and cost-effectiveness of a telehealth service delivering direct observation, compared to an in-person drive-around service. | VOT | In person DOT | Retrospective cohort study | Australia | AUD$2654 | AUD$2589 |  | $1.32 | AUD$1.32 (95% CI: $0.51 - $2.26) per extra day of successful observation |
| 3 | Holzman 2018 | To evaluate feasibility,accesibility and cost of VDOT when implemented under real world conditions | VDOT | Facility DOT | Pilot implementation study | USA | US$2065 | US$674 |  | -1391 | $1391 per patient for a standard 6-month treatment course |
| 4 | Salcedo 2021 | To evaluate the cost effectiveness of Aicure | VDOT | Facility DOT | Comparative study | USA | US$4894 | US$2668 |  | 4973 | VDOT treatment cost were $ 2.668 |
| 5 | Broomhead 2012 | The return on investment (ROI) for utilizing the SIMpill electronic treatment adherence solution as an adjunct to directly observed treatment short-course (DOTS) is assessed using data from a 2005 pilot of the SIMpill solution among new smear-positive tuberculosis (TB) patients in the Northern Cape Province. | EMM |  | cohort | South africa | |  |  |  | (US$ 493,221) for a cohort that would have started mid-2005 |
| 6 | Yang 2021 | To evaluate the cost-effectiveness of MEMS to monitor TB treatment among infectious active TB patients in Morocco. | EMM | Standard of care | Cohort Study | morroco | US$619 | US$745 |  | 434 |  |
| 7 | Hunchangsith 2012 | To evaluate the cost-effectiveness of different tuberculosis control strategies in Thailand. | FDOT | SAT | Economic study | Thailand | I$42M(25815 patients) | I$20M (25815 patients) | I$9400 | I$1000 dominant to I$1300 | (-I$9 million [95% uncertainty interval -I$12 million to -I$5 million]) |
|  |  |  | SMS |  |  |  | I$42M(25815 patients) | I$20M (25815 patients) | I$26000 | I$350 | (Incremental costs -9 (95% uncertainty interval -12 to -5)), |
| 8 | Nsengiyumva 2018 | To explore the costs and potential clinical impacts of the widely available and scalable technologies that are currently best positioned to replace in-person treatment observation either for active TB and/or to support treatment for LTBI that is currently self-administered | SMS | SAT | Decision analysis model | Brazil |  |  |  |  | incremental cost (95% UR) was USD 164 (USD 29 saving to USD 362 cost) per DALY averted |
| 9 | Datiko 2010 | To establish whether involving HEWs in TB control improved smear-positive case detection and treatment success rates in southern Ethiopia | CBDOT | HFDOT | Cluster randomized trial | Ethiopia | US$161.9 | US$60.9 |  | -16.3 | The cost per successfully treated patient was US$60.7 vs US$161.9 |
| 10 | KHAN 2002 | Assess effectiveness of different DOT strategies | CHW | SAT | RCT | Pakistan | US$164 | US$172 |  | US$239 |  |
| 11 | Adewole 2015 | To study the cost effectiveness and patients' satisfaction with home based direct observation of treatment (DOT), an innovative approach to community-based DOT (CBDOT) and hospital based DOT (HBDOT). | CBDOT | HFDOT | Randomized controlled trial | Nigeria | US$159.38 | US$89.52 |  | US$410 |  |
| 12 | PRADO 2011 | To compare the costs and outcomes associated with guardian-supervised directly observed treatment relative to the standard of care Directly Observed Therapy, Short Course (DOTS) provided by community health workers (CHW) | FDOT | CHW DOT | Comparative study | Brazil | US$398 | US4548 |  | -1,094.70 | Cost per patient treated with guardian-supervised DOTS was US$398, compared to US$548 for CHW-supervised DOTS. |

**Table 4:** List of excluded studies along with reasons for exclusion

Full-text articles excluded, with reasons (n=11)

Qualitative study (n= 1)

No exposure of interest (n= 7)

No outcome of interest (n= 3)

| **Study** | **Reason for Exclusion** |
| --- | --- |
| Dudley 2003 (1) | No exposure of interest |
| Bleumik 2001(2) | No exposure of interest |
| Singh 2004(3) | No exposure of interest |
| Barker 2002 (4) | No exposure of interest |
| Garfein 2018(5) | No outcome of interest  (Study examined adherence outcome) |
| Demaio 2001(6) | No outcome of interest  (Study examined adherence outcome) |
| Holzschuh 2017(7) | No outcome of interest  (Study examined adherence outcome) |
| Denkinger 2013(8) | Qualitative study |
| Kabongo 2010(9) | No exposure of interest |
| Zvavamwe 2009(10) | No exposure of interest |
| Niazi 2003(11) | No exposure of interest |

**References of excluded studies**

1. Dudley L, Azevedo V, Grant R, Schoeman JH, Dikweni L, Mahers D. Evaluation of community contribution to tuberculosis control in Cape Town, South Africa. Int J Tuberc Lung Dis. 2003;7(9 SUPPL. 1):48–55.

2. Becx-Bleumink M, Wibowo H, Apriani W, Vrakking H. High tuberculosis notification and treatment success rates through community participation in central Sulawesi, Republic of Indonesia. Int J Tuberc Lung Dis. 2001;5(10):920–5.

3. Singh AA, Parasher D, Shekhavat GS, Sahu S, Wares DF, Granich R. Effectiveness of urban community volunteers in directly observed treatment of tuberculosis patients: A field report from Haryana, North India. Int J Tuberc Lung Dis. 2004;8(6):800–2.

4. Barker RD, Millard FJC, Nthangeni ME. Unpaid community volunteers - Effective providers of directly observed therapy (DOT) in rural South Africa. South African Med J. 2002;92(4):291–4.

5. Garfein RS, Liu L, Cuevas-Mota J, Collins K, Muñoz F, Catanzaro DG, et al. Tuberculosis treatment monitoring by video directly observed therapy in 5 health districts, California, USA. Emerg Infect Dis. 2018;24(10):1806–15.

6. DeMaio J, Schwartz L, Cooley P, Tice A. The application of telemedicine technology to a directly observed therapy program for tuberculosis: A pilot project. Clin Infect Dis. 2001;33(12):2082–4.

7. Holzschuh EL, Province S, Johnson K, Walls C, Shemwell C, Martin G, et al. Use of Video Directly Observed Therapy for Treatment of Latent Tuberculosis Infection — Johnson County, Kansas, 2015. MMWR Morb Mortal Wkly Rep. 2017;66(14):387–9.

8. Denkinger CM, Grenier J, Stratis AK, Akkihal A, Pant-Pai N, Pai M. Mobile health to improve tuberculosis care and control: A call worth making. Int J Tuberc Lung Dis. 2013;17(6):719–27.

9. Kabongo D, Mash B. Effectiveness of home-based directly observed treatment for tuberculosis in Kweneng West subdistrict, Botswana. African J Prim Heal Care Fam Med. 2010;2(10).

10. Zvavamwe Z, Ehlers VJ. Experiences of a community-based tuberculosis treatment programme in Namibia: A comparative cohort study. Int J Nurs Stud. 2009;46(3):302–9.

11. Niazi AD, Al-Delaimi AM. Impact of community participation on treatment outcomes and compliance of DOTS patients in Iraq. East Mediterr Heal J. 2003;9(4):709–17.


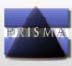
**PRISMA 2020 Checklist**

| **Section and Topic** | **Item #** | **Checklist item** | **Location where item is reported** |
| --- | --- | --- | --- |
| **TITLE** | | |  |
| Title | 1 | Identify the report as a systematic review. | I |
| **ABSTRACT** | | |  |
| Abstract | 2 | See the PRISMA 2020 for Abstracts checklist. | ii-iii |
| **INTRODUCTION** | | |  |
| Rationale | 3 | Describe the rationale for the review in the context of existing knowledge. | 1-3 |
| Objectives | 4 | Provide an explicit statement of the objective(s) or question(s) the review addresses. | 3 |
| **METHODS** | | |  |
| Eligibility criteria | 5 | Specify the inclusion and exclusion criteria for the review and how studies were grouped for the syntheses. | 5 |
| Information sources | 6 | Specify all databases, registers, websites, organisations, reference lists and other sources searched or consulted to identify studies. Specify the date when each source was last searched or consulted. | 3-4 |
| Search strategy | 7 | Present the full search strategies for all databases, registers and websites, including any filters and limits used. | 3-4 |
| Selection process | 8 | Specify the methods used to decide whether a study met the inclusion criteria of the review, including how many reviewers screened each record and each report retrieved, whether they worked independently, and if applicable, details of automation tools used in the process. | 4-5 |
| Data collection process | 9 | Specify the methods used to collect data from reports, including how many reviewers collected data from each report, whether they worked independently, any processes for obtaining or confirming data from study investigators, and if applicable, details of automation tools used in the process. | 7-8 |
| Data items | 10a | List and define all outcomes for which data were sought. Specify whether all results that were compatible with each outcome domain in each study were sought (e.g. for all measures, time points, analyses), and if not, the methods used to decide which results to collect. | 7-8 |
|  | 10b | List and define all other variables for which data were sought (e.g. participant and intervention characteristics, funding sources). Describe any assumptions made about any missing or unclear information. | 7-8 |
| Study risk of bias assessment | 11 | Specify the methods used to assess risk of bias in the included studies, including details of the tool(s) used, how many reviewers assessed each study and whether they worked independently, and if applicable, details of automation tools used in the process. | 6 |
| Effect measures | 12 | Specify for each outcome the effect measure(s) (e.g. risk ratio, mean difference) used in the synthesis or presentation of results. | 7 |
| Synthesis methods | 13a | Describe the processes used to decide which studies were eligible for each synthesis (e.g. tabulating the study intervention characteristics and comparing against the planned groups for each synthesis (item #5)). | 7-8 |
|  | 13b | Describe any methods required to prepare the data for presentation or synthesis, such as handling of missing summary statistics, or data conversions. | 7-8 |
|  | 13c | Describe any methods used to tabulate or visually display results of individual studies and syntheses. | 8-9 |
|  | 13d | Describe any methods used to synthesize results and provide a rationale for the choice(s). If meta-analysis was performed, describe the model(s), method(s) to identify the presence and extent of statistical heterogeneity, and software package(s) used. | 7-8 |
|  | 13e | Describe any methods used to explore possible causes of heterogeneity among study results (e.g. subgroup analysis, meta-regression). | 6 |
|  | 13f | Describe any sensitivity analyses conducted to assess robustness of the synthesized results. | 6 |
| Reporting bias assessment | 14 | Describe any methods used to assess risk of bias due to missing results in a synthesis (arising from reporting biases). | 6 |
| Certainty assessment | 15 | Describe any methods used to assess certainty (or confidence) in the body of evidence for an outcome. | 6 |
| **RESULTS** | | |  |
| Study selection | 16a | Describe the results of the search and selection process, from the number of records identified in the search to the number of studies included in the review, ideally using a flow diagram. | 8 |
|  | 16b | Cite studies that might appear to meet the inclusion criteria, but which were excluded, and explain why they were excluded. | 9 |
| Study characteristics | 17 | Cite each included study and present its characteristics. | 8-9 |
| Risk of bias in studies | 18 | Present assessments of risk of bias for each included study. | 9-10 |
| Results of individual studies | 19 | For all outcomes, present, for each study: (a) summary statistics for each group (where appropriate) and (b) an effect estimate and its precision (e.g. confidence/credible interval), ideally using structured tables or plots. | 10-19 |
| Results of syntheses | 20a | For each synthesis, briefly summarise the characteristics and risk of bias among contributing studies. | 10-19 |
|  | 20b | Present results of all statistical syntheses conducted. If meta-analysis was done, present for each the summary estimate and its precision (e.g. confidence/credible interval) and measures of statistical heterogeneity. If comparing groups, describe the direction of the effect. | 10-19 |
|  | 20c | Present results of all investigations of possible causes of heterogeneity among study results. | 9-10 |
|  | 20d | Present results of all sensitivity analyses conducted to assess the robustness of the synthesized results. | 9-10 |
| Reporting biases | 21 | Present assessments of risk of bias due to missing results (arising from reporting biases) for each synthesis assessed. | 9-10 |
| Certainty of evidence | 22 | Present assessments of certainty (or confidence) in the body of evidence for each outcome assessed. | 14-19 |
| **DISCUSSION** | | |  |
| Discussion | 23a | Provide a general interpretation of the results in the context of other evidence. | 20-22 |
|  | 23b | Discuss any limitations of the evidence included in the review. | 22 |
|  | 23c | Discuss any limitations of the review processes used. | 22 |
|  | 23d | Discuss implications of the results for practice, policy, and future research. | 22 |
| **OTHER INFORMATION** | | |  |
| Registration and protocol | 24a | Provide registration information for the review, including register name and registration number, or state that the review was not registered. | 3 |
|  | 24b | Indicate where the review protocol can be accessed, or state that a protocol was not prepared. | 3 |
|  | 24c | Describe and explain any amendments to information provided at registration or in the protocol. | Refer 3 |
| Support | 25 | Describe sources of financial or non-financial support for the review, and the role of the funders or sponsors in the review. | 24 |
| Competing interests | 26 | Declare any competing interests of review authors. | 24 |
| Availability of data, code and other materials | 27 | Report which of the following are publicly available and where they can be found: template data collection forms; data extracted from included studies; data used for all analyses; analytic code; any other materials used in the review. | 24 |

*From:*  Page MJ, McKenzie JE, Bossuyt PM, Boutron I, Hoffmann TC, Mulrow CD, et al. The PRISMA 2020 statement: an updated guideline for reporting systematic reviews. BMJ 2021;372:n71. doi: 10.1136/bmj.n71

For more information, visit: <http://www.prisma-statement.org/>

**Search strategy and selection criteria**

The following databases, Medline/PubMed, EBSCO (PsycINFO and CINAHL), Cochrane libraries EMBASE, WHO, grey literature, were searched using free text and controlled vocabulary terms (MeSH) for studies published from January 2000 till September 2022. The PICO framework informed search terms (Table 5).

The final search strategy included the following: (“tuberculosis, community” [MeSH Terms] OR (“Tuberculosis” [Text Word] OR “TB” [Text Word])) AND 01/01/00:2022/09/30 [Date—Publication] AND (“treatment completion” [Title/Abstract] OR “treatment cure” [Title/Abstract] OR “treatment success”[Title/Abstract] OR “Cost effectiveness”[Title/Abstract]

**Table 5. Detail of PICO components that informed search strategy.**

| **PICO** | |
| --- | --- |
| **P** | Individuals living with TB |
| **I** | Operational interventions |
| **C** | Standard-of-care |
| **O** | TB treatment outcomes and model viability |

*Population*: Tuberculosis OR TB

*Intervention*: Intervention; community-based DOT, family DOT, electronic medication reminders, video observed treatment

*Outcome*: treatment completion, treatment cure, treatment success, cost effectiveness.

We also searched through the reference lists of similar published systematic reviews to identify

studies not captured by our database search outcomes. Details of search outcomes are in

Table B 6

| Table 6: Search Strategy and Results for Each Database Searched | | |
| --- | --- | --- |
| PubMed | | |
| **Search** | **Query** | **Results** |
| 1 | "Tuberculosis, community"[MeSH Terms] OR "Tuberculosis" OR "TB" | 29,702 |
| 2 | Treatment completion OR completed treatment OR treatment cure OR cured OR treatment success OR successful outcome Or Cost effectiveness OR cost saving OR incremental cost effectiveness | 50,867 |
| 3 | (Intervention OR interventions OR interventional OR cohort* OR trial OR trials OR RCT): | 51,417 |
| #4 | #1 AND #2 AND #3 | 2,444 |
| Embase | | |
| **1** | Tuberculosis or TB | 42,377 |
| **2** | Treatment completion OR completed treatment OR treatment cure OR cured OR treatment success OR successful outcome Or Cost effectiveness OR cost saving OR incremental cost effectiveness | 37,198 |
| **3** | (Intervention OR interventions OR interventional OR cohort* OR trial OR trials OR RCT): | 59,297 |
| **4** | #1 AND #2 AND #3 | 3,596 |
| CINAHL | | |
| **S1** | Tuberculosis or TB | 1,047 |
| **S2** | Treatment completion OR completed treatment OR treatment cure OR cured OR treatment success OR successful outcome Or Cost effectiveness OR cost saving OR incremental cost effectiveness | 14,100 |
| **S3** | (Intervention OR interventions OR interventional OR cohort* OR trial OR trials OR RCT): | 16,846 |
| **S4** | S1 AND S2 AND S3 | 1,955 |
| PsycINFO | | |
| **S1** | Tuberculosis or TB | 5,059 |
| **S2** | Treatment completion OR completed treatment OR treatment cure OR cured OR treatment success OR successful outcome Or Cost effectiveness OR cost saving OR incremental cost effectiveness | 74,566 |
| **S3** | (Intervention OR interventions OR interventional OR cohort* OR trial OR trials OR RCT): | 61,038 |
| **S4** | S1 AND S2 AND S3 | 209 |
| **Cochrane Trials** | | |
| **S1** | Tuberculosis or TB | 594 |
| **S2** | Treatment completion OR completed treatment OR treatment cure OR cured OR treatment success OR successful outcome Or Cost effectiveness OR cost saving OR incremental cost effectiveness | 63,390 |
| **S3** | (Intervention OR interventions OR interventional OR cohort* OR trial OR trials OR RCT): | 44,756 |
| **S4** | S1 AND S2 AND S3 | 1327 |
